# In Silico Ligand-Based Screening of PDB Database for Searching Unique Motifs Against SARS-CoV-2

**DOI:** 10.3390/biom16010163

**Published:** 2026-01-19

**Authors:** Andrey V. Machulin, Juliya V. Badaeva, Sergei Y. Grishin, Evgeniya I. Deryusheva, Oxana V. Galzitskaya

**Affiliations:** 1 G.K. Skryabin Institute of Biochemistry and Physiology of Microorganisms, Federal Research Center Pushchino Scientific Center for Biological Research, Russian Academy of Sciences, 142290 Pushchino, Russia; and.machul@gmail.com; 2 Moscow Timiryazev Agricultural Academy, Russian State Agrarian University, 127434 Moscow, Russia; jushipper@gmail.com; 3 Institute of Protein Research, Russian Academy of Sciences, 142290 Pushchino, Russia; syugrishin@gmail.com; 4 Institute for Biological Instrumentation, Federal Research Center Pushchino Scientific Center for Biological Research, Russian Academy of Sciences, 142290 Pushchino, Russia; janed1986@ya.ru; 5 Gamaleya National Research Center for Epidemiology and Microbiology, 123098 Moscow, Russia; 6 Institute of Theoretical and Experimental Biophysics, Russian Academy of Sciences, 142290 Pushchino, Russia

**Keywords:** severe acute respiratory syndrome coronavirus 2 (SARS-CoV-2), angiotensin converting enzyme II (ACE2), receptor-binding domain (RBD), binding motifs, therapeutic antibodies, PDB database

## Abstract

SARS-CoV-2, the virus responsible for coronavirus disease COVID-19, is a highly transmissible pathogen that has caused substantial global morbidity and mortality. The ongoing COVID-19 pandemic caused by this virus has had a significant impact on public health and the global economy. One approach to combating COVID-19 is the development of broadly neutralizing antibodies for prevention and treatment. In this work, we performed an in silico ligand-based screening of the PDB database to search for unique anti-SARS-CoV-2 motifs. The collected data were organized and presented in a classified SARS-CoV-2 Ligands Database, categorized based on the number of ligands and structural components of the spike glycoprotein. The database contains 1797 entries related to the structures of the spike glycoprotein (UniProt ID: P0DTC2), including both full-length molecules and their fragments (individual domains and their combinations) with various ligands, such as angiotensin-converting enzyme II and antibodies. The database’s capabilities allow users to explore various datasets according to the research objectives. To search for motifs in the receptor-binding domain (RBD) most frequently involved in antibody binding sites, antibodies were classified into four classes according to their location on the RBD; for each class, special binding motifs are revealed. In the RBD binding sites, specific tyrosine-containing motifs were found. Data obtained may help speed up the creation of new antibody-based therapies, and guide the rational design of next-generation vaccines.

## 1. Introduction

Severe acute respiratory syndrome coronavirus 2 (SARS-CoV-2) contains a phosphorylated nucleocapsid protein enclosing a single-stranded RNA genome. This ribonucleoprotein complex is surrounded by a phospholipid bilayer envelope, forming spherical or pleomorphic virions with diameters ranging from 80 to 120 nm, distinguished by prominent surface spike (S) glycoproteins [1,2]. The S glycoprotein, a type I transmembrane protein that is extensively glycosylated, is anchored in the viral envelope [3]. It comprises two main subunits: S1 and S2. The S1 subunit mediates the recognition of host cell receptors essential for viral attachment, while the S2 subunit facilitates the fusion between viral and cellular membranes, promoting viral entry. The boundary between S1 and S2 contains a unique furin cleavage site processed by the host furin protease, which enhances the infectivity of the virus [4].

SARS-CoV-2 utilizes the angiotensin-converting enzyme 2 (ACE2) receptor, expressed in many human tissues, as its primary entry point into cells. Binding to ACE2 exposes a secondary S2′ cleavage site, which is subsequently processed by transmembrane serine protease 2 (TMPRSS2) on the cell surface, mediating fusion of the viral and cellular membranes [5]. Alternatively, ACE2-bound virions can be internalized via endocytosis, in which the cleavage of the S2′ site is catalyzed by lysosomal cathepsins, particularly cathepsin L [6,7].

**Figure 1:**
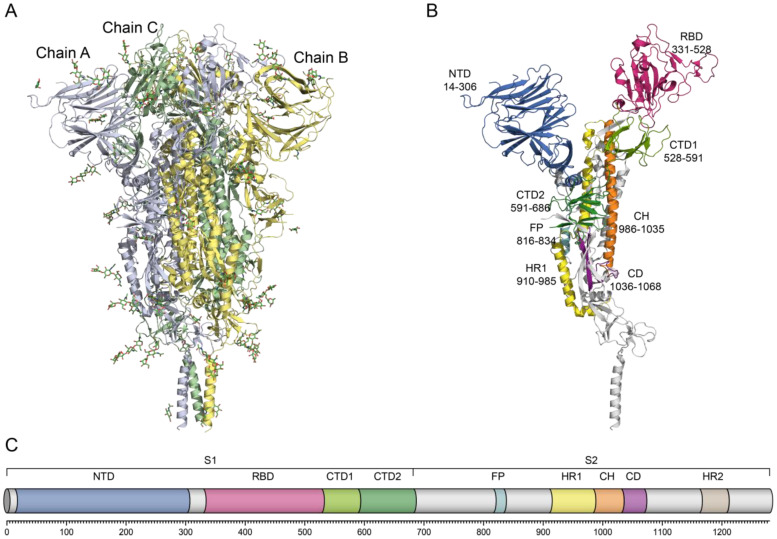
The molecular structure of SARS-CoV-2 S protein (UniProt ID P0DTC2, 1273 a.a. residues). (**A**) Prefusion conformation of the homotrimer SARS-CoV-2 (PDB ID: 6XR8, EM). (**B**) Location of structural domains in one subunit of SARS-CoV-2 S protein. NTD: N-terminal domain (blue), RBD: receptor-binding domain (rose), CTD1: C-terminal domain 1 (light green), CTD2: C-terminal domain 2 (dark green), FP: fusion peptide (cyan), HR1: heptad repeat 1 (yellow), CH: central helix region (orange), CD: connector domain (purple). Borders of structural domains are indicated. HR2, TM and CT domains were not seen in the crystal structure of the trimeric S protein ectodomain (PDB ID: 6XR8). (**C**) Schematic representation of the domain location of the SARS-CoV-2 S protein. S1/S2: S1/S2 furin cleavage site.

The S1 subunit (residues 1–685) comprises the N-terminal domain (NTD, 14–306), the receptor-binding domain (RBD, 331–528), and the C-terminal domains (CTD1, 528–591; CTD2, 591–686). The S2 subunit (residues 686–1273) contains the fusion peptide (FP, 816–855), heptad repeat 1 (HR1, 918–983), and heptad repeat 2 (HR2, 1163–1203), each of which is critical for mediating membrane fusion (Figure 1). The NTD of the spike protein is organized into three structural regions decorated with eight N-linked glycans, which is similar to the NTDs of other coronaviruses. Although its exact biological function remains controversial, in related viruses, the NTD is known to be involved in carbohydrate or protein receptor binding and conformational rearrangements [8].

The RBD plays a central role in virus–host interaction because it directly contacts the ACE2 receptor on human cells. This binding occurs through the receptor-binding motif (RBM), and specific mutations (e.g., N501Y, K417N, E484K) are known to increase affinity for ACE2, thereby enhancing viral transmissibility and immune evasion potential. Due to its important role in entry, the RBD serves as a primary target for neutralizing antibodies, which can block interaction with ACE2 or inhibit conformational changes in the spikes required for fusion. Therapeutic approaches using soluble ACE2 and its multivalent derivatives have shown potent neutralizing effects in vitro by competitively binding the RBD, thereby preventing viral attachment to host cells [9].

CTDs of S protein are predominantly β-structured and span both S1 and the adjacent N-terminus of S2 near the furin site. CTD1 contains two antiparallel β-sheets (with two and four strands), whereas CTD2 consists of two four-stranded β-sheets including one of S2 [9]. In the RBD–down conformation of trimeric S protein, the “630 loop” of CTD2 is ordered in the G614 variant but disordered in the ancestral Wuhan-Hu-1 strain. This loop stabilizes CTD2 by inserting between the NTD and CTD1 within a single protomer, located close to the S1/S2 junction and the fusion peptide proximal region (FPPR) of the adjacent protomer. The concerted movement of FPPR and 630 loop regulates the transition of RBD between the up and down states, thereby controlling the conformational dynamics required for membrane fusion [9].

Following furin cleavage at the S1/S2 site, the S1 and S2 subunits remain noncovalently attached, maintaining the ability to fuse. A subsequent proteolytic event at the S2′ site (R815), mediated by TMPRSS2 or endosomal cathepsins, releases a hydrophobic FP (residues 816–834), which embeds into the host membrane, initiating fusion. Structurally, FP consists of two fragments: residues S816–V826 form a short coiled-coil structure within a negatively charged pocket, while residues T827–I834 represent a flexible loop located in a positively charged cavity [10]. In the prefusion state, the HR1 domain (G910–D985) forms four α-helices. Upon transition to the post-fusion conformation, HR1 rearranges into an extended central three-stranded coiled-coil (>180 Å) in association with the CH domain [9]. HR2 (D1163–K1211) binds antiparallel to HR1 via hydrophobic and hydrogen bonds, producing a six-helix bundle (6-HB) that drives membrane fusion. Due to their critical roles, HR1 and HR2 are recognized as attractive targets for the development of fusion inhibitors and vaccines [10]. The connector domain (CD), together with residues 718–729 from the S1/S2–S2′ region, forms a three-stranded β-sheet. Residues 1127–1135 extend this sheet to four strands, while the segment spanning residues 737–769 forms three α-helices linked by two disulfide bonds. These helices interact with the CH groove to form 6HB-1 connecting CH to HR2 via a flexible linker [10].

Antibodies that target viral pathogens are classified as neutralizing (nAbs) and non-neutralizing (non-nAbs). Neutralizing antibodies block viral entry into cells or membrane fusion, thereby preventing infection, while non-nAbs lack direct neutralizing activity but can mediate immune defense via Fc-dependent mechanisms, such as antibody-dependent cellular cytotoxicity, phagocytosis, or complement activation. For SARS-CoV-2, nAbs-recognizing epitopes within the NTD and RBD regions of the S1 domain, as well as the stem helix (SH) and FP regions of the S2 domain, have been described in details [11].

NTD-directed nAbs isolated from COVID-19 convalescents predominantly target two glycan-free antigenic sites, NTD-1 and NTD-2. Most of them interact with the NTD-1 “supersite,” comprising five surface loops and a β-structure that is rearranged upon antibody binding. Mutations and deletions in this region, characteristic of variants such as Alpha, Beta, Gamma, and Delta, reduce sensitivity to neutralization by these antibodies [9].

The discovery of therapeutic agents capable of inhibiting the key proteins of SARS-CoV-2 remains a critical challenge. To facilitate this process, the integration and standardization of experimental and computational data on the interactions of viral proteins with ligands is crucial. The creation of specialized SARS-CoV-2 ligand database [12] allows for the consolidation of structural, biochemical, and pharmacological information, providing a powerful tool for drug discovery and in silico modeling. Such databases enhance the efficiency of identifying and optimizing molecules with high binding affinity to viral targets, accelerating the development of safe and effective antiviral drugs against COVID-19.

Currently, there are many specialized data resources on SARS-CoV-2: GISAID (https://www.gisaid.org, accessed on 8 December 2025) is a global platform for sharing viral genome sequences used to monitor mutation patterns and evolutionary trends; Outbreak.info (https://outbreak.info, accessed on 8 December 2025) allows for the geographic tracking of variants and literature mining; CoV-Spectrum (https://cov-spectrum.org, accessed on 8 December 2025) supports rapid comparison of variant prevalence and mutation profiles; COVID-19 CG (http://covidcg.org, accessed on 8 December 2025) facilitates visualization of mutation frequencies and global distribution; the Coronavirus Antiviral & Resistance Database (CoV-RDB) (https://covdb.stanford.edu, accessed on 8 December 2025) is a curated repository of antiviral resistance data from Stanford University; EMBL-EBI COVID-19 Data Platform (https://www.covid19dataportal.org/, accessed on 8 December 2025) offers integrated tools for storing, analyzing, and visualizing SARS-CoV-2 data across Europe.

A number of databases and community resources have been developed to support SARS-CoV-2 research, encompassing experimental structures, modeling results, visualization platforms, and integrative data portals (e.g., PDBe COVID-19; covid-19.bioreproducibility.org, accessed on 8 December 2025; coronavirus_structural_task_force; coronavirus3d.org, accessed on 8 December 2025; COVID-19 Data Portal, among others). In the present study, the overview of the related resources is intentionally selective rather than exhaustive. We focus specifically on databases that provide curated, experimentally determined structures of SARS-CoV-2 spike protein complexes and enable systematic, residue-level analysis of spike–ligand interactions. While several complementary resources primarily emphasize data aggregation, visualization, or reproducibility frameworks, the interaction-centric scope of this work is designed to address a distinct need: the structured comparison of experimentally resolved spike–ligand interfaces. This focus defines the conceptual framework and intended use of the database introduced here. In this work, we collected all available SARS-CoV-2 Ligand structures from the PDB and created a dedicated database for them. To identify unique molecular motifs capable of effectively targeting key SARS-CoV-2 proteins, the SARS-CoV-2 ligand complexes were analyzed. The identified motifs represent promising candidates for further experimental validation and drug development. Ultimately, this study will help accelerate the discovery of effective therapeutic strategies against COVID-19.

## 2. Materials and Methods

### 2.1. Construction and Analysis of SARS-CoV-2-Antibodies Dataset

A dataset containing structural complexes of SARS-CoV-2 antibodies was compiled from the Protein Data Bank (PDB; https://www.rcsb.org, accessed on 8 December 2025) [13]. The selection was based on entries corresponding to the amino acid sequence of the Spike glycoprotein (UniProt ID: P0DTC2). Data retrieval, processing, and analytical operations were implemented in the Python 3 programming environment (https://www.python.org/, accessed on 8 December 2025), executed through the PyCharm v.2018 integrated development environment. To determine inter-residue interactions, pairs of contacting amino acids were identified as those with Cα-Cα distances of ≤5 Å. The Cα-Cα distance threshold was chosen as a balance between the higher specificity associated with lower heavy atom contact cutoffs (approximately ~3–4.5 Å) [14] and the increased sensitivity obtained with larger distance thresholds commonly employed in backbone-based structural analyses (≤7–8 Å) [15,16,17]. The calculations were performed using a customized Python script within the PyMOL molecular visualization package (v.2.5.0; https://pymol.org/2/, accessed on 8 December 2025).

The identified contact residues in antibody–spike complexes were subsequently mapped to their respective amino acid sequences according to the following steps. Sequence alignments were generated with Clustal Omega [18] (https://www.ebi.ac.uk/jdispatcher/msa/clustalo, accessed on 8 December 2025). Sequence alignments result is shown at list “all” of the Supplementary Table S1. In the resulting alignment, each RBD sequence is highlighted in red and marked “*”. For each amino acid residue, the amount of its entry into the antibody binding site for the studied group of complexes was calculated (“amount”, Supplementary Table S1). After dividing the antibodies into four classes, the relative amount of each amino acid residue in the antibody binding sites was calculated for each group. Three-dimensional molecular representations were rendered using PyMOL v.2.5.0.

### 2.2. Database Development

The database infrastructure was constructed using Structured Query Language (SQL) within the MySQL database management system (DBMS; https://www.mysql.com/, accessed on 8 December 2025). MySQL Workbench (https://www.mysql.com/products/workbench/, accessed on 8 December 2025) was utilized for data handling and graphical visualization.

A web interface was designed to provide user-friendly access to the database. The web application was developed in the Visual Studio Code, version 1.108 environment (https://code.visualstudio.com/, accessed on 8 December 2025) using standard front-end technologies-HTML, CSS, and JavaScript. The data tables were exported from SQL format and integrated into the web framework in HTML format via MySQL Workbench tools.

## 3. Results

### 3.1. Database Presentation

The database structure consists of 14 tables: FULL (977 records), FULL-TM (79 records), NTD-RBD-CTD1-CTD2 (5 records), CTD1-CTD2-CH-CD-SH-HR2 (1 record), NTD (14 records), RBD (628 records), CTD2 (2 records), FP (11 records), CH (14 records), HR1 (24 records), HR2+TM (37 records), CD (6 records), FP-HR1 (1 record), and FP-HR1-CH-CD-SH-HR2-TM (9 records). The data in these tables were extracted from the main POLY table, which serves as the source of information from the PDB database. Each table contains several columns, including the following: ID, PDB ID, Method, Resolution, Chain, Start, End, Macromolecules, and Ligand. The number of Ligands column varies from 1 to 8 depending on the specific table. The ID field is of type INT, does not allow for null values, and is designated as the primary key with auto-increment. The PDB ID field is of type VARCHAR and can contain values ranging from 100 to 200 characters in length, also disallowing null values. The fields Method, Resolution, Chain, Macromolecules, and Ligand also have a VARCHAR type with lengths ranging from 100 to 200 characters. The Start and End fields are of type INT.

**Figure 2:**
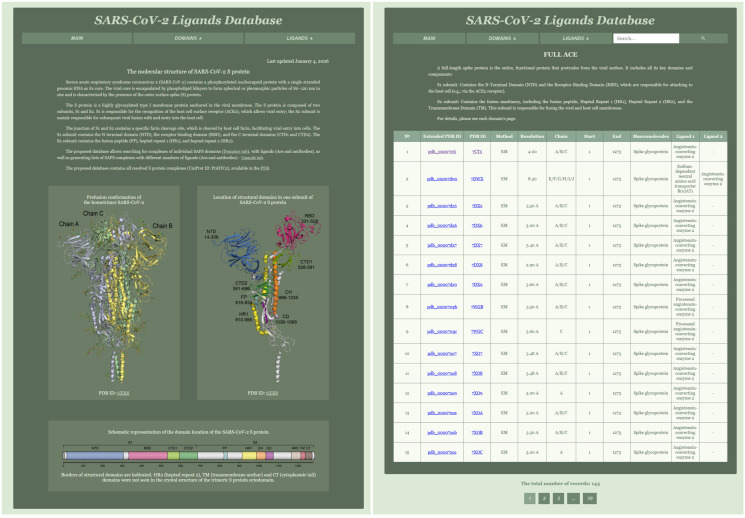
(**Left**) Main page of the website containing information about the molecular structure of SARS-CoV-2 and main tabs; (**right**) page of DOMAIN RBD containing information about its ligands with features (PDB ID, Method, Resolution, Chain/chains corresponding to SARS-CoV-2, borders of RBD domain, list of the ligands).

The website consists of three tabs: MAIN, DOMAINS, and LIGANDS. The MAIN tab serves as the homepage and contains information about the molecular structure of the SARS-CoV-2 S protein, including three images (Figure 2).

The DOMAINS tab features a dropdown menu with the names of all database tables, where each page contains the corresponding table and a brief description. The tables have horizontal scrolling for ease of viewing. The LIGANDS tab includes a dropdown menu with the number of ligands. Additionally, the website incorporates a search bar that allows users to search for specific records within the tables.

### 3.2. Distribution of SARS-CoV-2-Ligands Structures

Of the spike glycoprotein, 1797 structures (UniProt ID: P0DTC2) have been deposited in the Protein Data Bank (PDB), including both full-length molecules and their fragments (individual domains and their combinations). All structures were classified into groups according to unique domains or their combinations (Table 1).

The predominant number of submitted structures is full-length proteins (977 structures) and RBD (628 structures). Most of the available structures were obtained using electron microscopy (EM) (1303 structures). The structure of the S protein is mainly represented as a homotrimer (916 structures). The analysis of ligands (Fab fragments of antibodies and ACE2) showed that their number varies: 455 structures contain one ligand, 766 structures contain two ligands, and 192 structures contain three or more ligands.

Additionally, for each structure, information was collected on the low-molecular-weight ligands that make up the complex, among which zinc ions and saccharides (alpha-D-mannose and 2-acetamido-2-deoxy-beta-D-glucopyranose) predominate.

### 3.3. RBD Domain and Its Ligands

**Figure 3:**
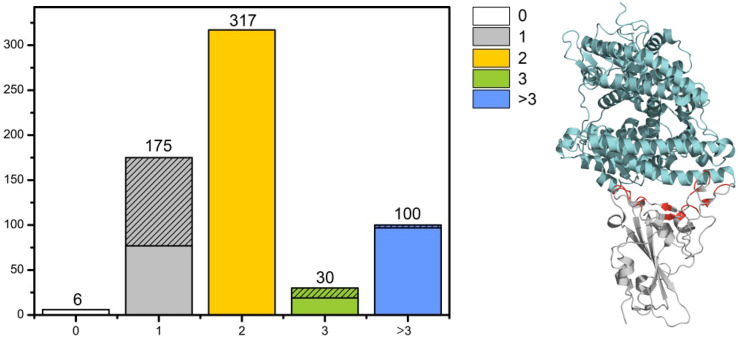
(**Left**) Distribution of the RBD-ligands complexes. Shaded areas correspond to complexes with ACE as ligand; (**right**) example of RBD (cyan)—ACE2 (grey) complex structure (PDB ID: 6M0J). The receptor-binding motif (RBM, red) (part of the hypervariable loop located within the RBD containing the amino acids that recognize the ACE2) revealed using Pymol script (Cα-Cα distances of ≤5 Å) contain residues K417, G446, G447, Y449, Y453, L455, F456, Y473, A475, G476, E484, F486, N487, Y489, Q493, G496, Q498, T500, N501, G502, Y505.

The RBD group (628 structures) was isolated as a separate sample for the primary analysis. The RBD domain structure (both with and without ligands) is mainly represented as a monomer (446 structures). This group is dominated by structures with one (175 structures) and two (317 structures) ligands (Fab fragments of antibodies and ACE2). In the group with one ligand, 99 structures correspond to complexes RBD-ACE2 and in the group with three ligands 11 structures correspond to complexes RBD-ACE2 (Figure 3 Left).

**Figure 4:**
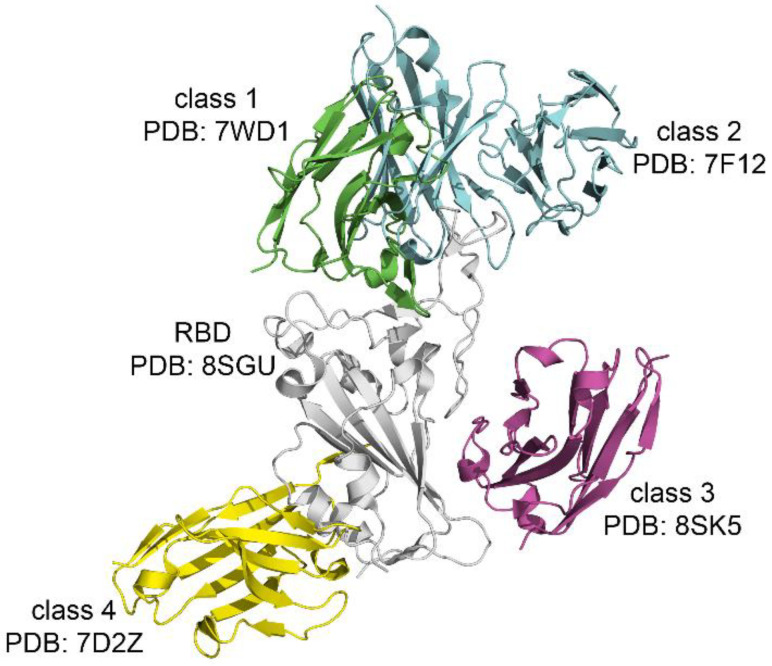
RBD–directed antibodies divided into four main classes depending on the epitopes they target in the RBD of the S protein.

Most anti-SARS-CoV-2 antibodies target the RBD and can be classified into different groups based on their specific epitopes. The most widely used classification, proposed in reference [19], divides RBD-targeting antibodies into four classes according to their mode of binding to the S protein (Figure 4).

Using data obtained (http://oka.protres.ru/SARS-CoV-2Ligands/, accessed on 8 December 2025) RBD–antibody complexes (one ligand), structures were classified into four classes according location of antibody on RBD (Supplementary Table S1). According to the data obtained, the most represented antibodies are those belonging to class 1 (41 structures). Class 2 includes 4 structures, the third class includes 2, and the 4th class includes 9 structures. Some antibodies cannot be assigned to a separate class since they are located in the areas of several classes at the same time (Supplementary Table S1).

The epitope recognized by class 1 antibodies should overlaps with RBM within the RBD. Their main neutralization mechanism involves blocking the binding of ACE2 to the S protein. Class 2 antibodies also target the RBM in the area near to the class 1. Antibodies in classes 3 and 4 bind to the RBD but do not directly inhibit ACE2-RBD interaction. It is possible that the action of class 3 and 4 antibodies is associated with steric hindrance between RBD and ACE2 [19].

### 3.4. Analysis of Amino Acid Composition of Binding Sites

**Figure 5:**
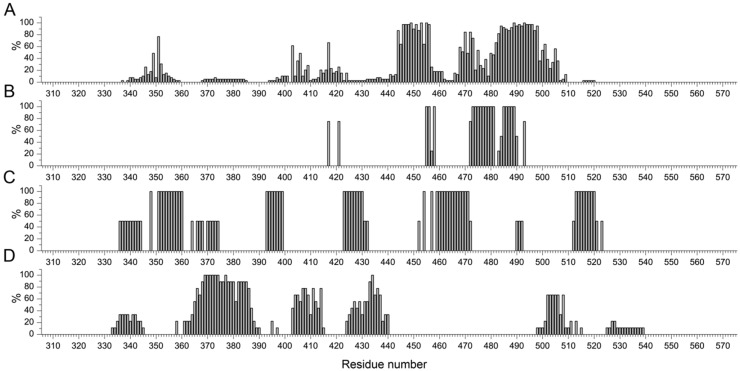
Distribution of the relative content of each amino acid residue in the binding sites of RBD with antibodies (one chain/single name of ligand) divided into four classes: (**A**) 1, (**B**) 2, (**C**) 3, (**D**) 4 (only for records clearly belonging to the same class, Supplementary Table S1).

Multiple alignments of the primary structures of the RBD domains (using Clustal O) from the studied complexes of the group has been performed, which showed the presence of a number of mutations in some structures (Supplementary Table S1). The distribution of binding site residues in RBD for the four classes of antibodies is shown in Figure 5.

To search for motifs in RBD most frequently involved in antibody binding sites, residues for each of the 4 classes were selected that were involved in interactions for more than 50 percent of the entries in the group. As a result, the following motifs are revealed:

- for class 1: Y351, R403, K417, 444KVGGN**YNYL**YRLF456, T468, S469, T470, I472, Y473, A475, 482GVEGFNC**YFPL**QSYGFQ498, T500, N501, Y505;
- for class 2: K417, Y421, L455, F456, K458, 472 I**YQAG**STPCN481, 485GFNCY489, Q493;
- for class 3: A348, 351YAWNRKRISN360, 393TNVYADS399, 423**YKLP**DDFT430, R454, R457, 459SNLKPFERDISTE471, 513LSFELLHA520;
- for class 4: 365**YSVL**YNSASFSTFKCYGVSPTK386, 404GDEVRQ409, A411, P412, Q414, D427, F429, 431GCVIAWN437, 502GVGYQ506, Y508.

For classes 1 and 2, the regions of the “accepted” RBM motif (G446, G447, Y449, Y453, L455, F456, Y473, A475, G476, E484, F486, N487, Y489, Q493, G496, Q498, T500, N501, G502, Y505) are included in the binding regions identified by us with the addition of a number of other amino acid residues. It is also evident that some of the residues in classes 3 and 4 “capture” the binding regions with ACE, which confirms the assumption about the mechanism of their action, namely partial steric competition for binding.

**Figure 6:**
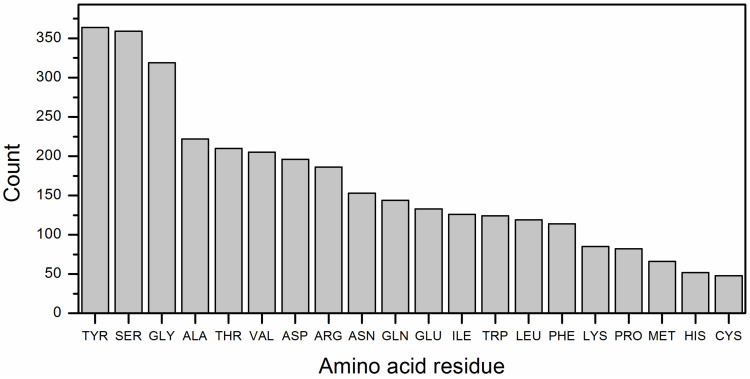
Distribution of the content of each amino acid residue in the binding sites of antibodies (one chain/single name of ligand, 76 records) with RBD.

The distribution of amino acid residues in antibodies (one chain/ single name of ligand) that are part of the binding sites with RBD is shown in Figure 6. In single-chain antibodies the interaction with RBD occurs mainly through Y, S and G.

Among class 1 antibody ligands (one chain/ single name of ligand), 10 entries correspond to broadly neutralizing antibodies (Supplementary Table S1). Analysis of the distribution of binding residues on the RBD revealed the following binding motif: Y351, R403, E406, S408, Q409, Q409, N417, 444KVGGN**YNYL**YRLFR457, 468IST470, I472, Y473, A475, G476, K478, 483VAGFNC**YFPL**RSYGFR498, T500, Y501.

## 4. Discussion

The explosive growth of structural studies on the SARS-CoV-2 Spike glycoprotein during the COVID-19 pandemic has created a fragmented but extremely rich body of structural information [20,21]. Although nearly two thousand spike-related entries have been deposited in the Protein Data Bank, these structures are dispersed across different domains, ligand types, experimental methods, and complex study designs. As a result, researchers face significant difficulties when attempting to perform comparative analyses, identify recurring ligand–binding patterns, or locate structures corresponding to specific spike regions [22]. The SARS-CoV-2 Ligands Database developed in this work directly addresses this problem by providing a systematically organized, domain-oriented, searchable platform that integrates 1797 spike structures and their ligands into a consistent framework.

A key contribution of this work is the classification of all available spike structures according to their position along the polypeptide chain, resulting in 14 domain-specific groups that cover the full-length Spike (1–1273 a.a. residues), as well as its constituent functional regions: NTD, RBD, CTD2, FP, CH, HR1, HR2, CD, TM, and combinations thereof. The domain boundaries are clearly presented on the website (Figure 2, left panel), ensuring transparency and reproducibility. Each structural group is linked to extensive metadata extracted from the PDB via the consolidated POLY table, including PDB ID, experimental method (e.g., X-ray diffraction, cryo-EM), chain identifiers corresponding to SARS-CoV-2, domain start and end positions, macromolecular composition, and ligand lists. This structured format enables users to retrieve information that would otherwise require manual inspection of individual PDB entries [23,24].

The web interface plays a central role in making the dataset accessible. The MAIN page provides essential biological context about the molecular architecture of the Spike protein, accompanied by high-quality structural figures that illustrate domain organization and trimeric assembly. The DOMAINS tab enables users to navigate directly to each of the 14 structural groups and view the corresponding tables, each enriched with domain descriptions and horizontal scrolling for convenient inspection of large datasets. The RBD domain page exemplifies this functionality: it contains a sortable table with 628 entries, including details on ligands such as ACE2, Fab fragments of neutralizing antibodies, nanobodies, and ions, together with experimental metadata that allow users to distinguish between high-resolution crystallographic structures and lower-resolution cryo-EM complexes. Equivalent domain-resolved views are provided for all other spike regions, enabling fine-grained comparative analysis across the full structural landscape of the glycoprotein.

The use of a Cα-Cα distance threshold of ≤5 Å represents a methodological compromise between specificity and sensitivity in defining residue–residue proximity across a heterogeneous set of spike–ligand complexes. Compared with conventional heavy atom contact definitions (typically ≤4–4.5 Å), a Cα-based criterion inevitably sacrifices atomic-level precision and may include residues that do not form direct physicochemical interactions. However, stricter heavy atom cutoffs can be overly sensitive to local side-chain conformations, coordinate uncertainty, and resolution differences between X-ray crystallography and cryo-EM structures, potentially leading to underestimation of recurring interaction patterns. Conversely, broader backbone-based distance thresholds (e.g., >7 Å) increase sensitivity but may incorporate residues that are only indirectly associated with the interaction interface [25]. The selected ≤5 Å Cα-Cα cutoff, therefore, reflects a pragmatic balance, capturing structurally conserved proximity patterns while limiting noise arising from structural variability. The same ≤5 Å threshold between heavy atoms was used for protein–interface clustering in a study identifying “sub-interfaces” in protein-protein complexes [26]. Nevertheless, the resulting motifs should be interpreted as indicators of recurrent spatial neighborhoods rather than definitive atomic contacts, and their biological relevance must be considered in the context of complementary biochemical and structural evidence.

The distribution of ligands across domains provides several insights into the structural biology of SARS-CoV-2. As expected, full-length spike structures (977 entries) and RBD structures (628 entries) dominate the dataset, reflecting the intense research focus on receptor recognition, neutralizing epitopes, and conformational dynamics of the prefusion spike. The diversity of ligands, ranging from antibody fragments and ACE2 to saccharides and zinc ions, mirrors the multifunctional nature of the spike protein and the wide range of biochemical strategies used to stabilize, inhibit, or probe its structure. The classification presented in Table 1 highlights substantial structural heterogeneity: 454 structures contain one ligand, 755 contain two ligands, and 152 contain three or more ligands, illustrating the complexity of multivalent engagement within the spike architecture.

Importantly, the database not only aggregates structural records but also provides a foundation for downstream comparative and analytical work. The detailed annotation of RBD structures, for example, reveals the dominance of monomeric formats and highlights recurrent binding modes of ACE2 and neutralizing antibodies. By cataloguing all molecules in the complex (except low-molecular-weight substances) as ligands, the database offers a comprehensive landscape of interacting partners for each spike region, which in turn facilitates further functional analysis of spike variants and their ligands. The availability of domain-resolved ligand lists and metadata supports further analyses such as classification of antibody-binding epitopes, structural alignment of recurring ligand footprints, clustering of ligand-binding motifs, and assessment of domain-specific structural conservation [27,28]. The database, therefore, establishes a platform that bridges raw structural data with hypothesis-driven research on immune recognition, viral evolution, and therapeutic design.

From a methodological standpoint, the systematic organization of nearly two thousand spike structures into clearly defined domain groups represents a significant contribution to the field. Previous structural surveys of SARS-CoV-2 have typically focused on specific subsets [29], such as RBD-ACE2 complexes [30] or antibody classes [19], whereas our resource encompasses the entire Spike protein and all associated ligands identified to date. By embedding this information into a user-friendly web interface with search capabilities and hierarchical navigation, the database provides a centralized tool that can support comparative analyses, facilitate reproducibility, and accelerate the identification of structural patterns relevant to vaccine and therapeutic development [31].

Previous computational studies have demonstrated the critical importance of high-quality structural reference data for understanding SARS-related biomolecules and for developing, benchmarking, and validating predictive modeling approaches [32,33]. Although neither of these studies directly addresses protein-ligand or antibody–spike interactions, they collectively emphasize the importance of curated, experimentally grounded structural datasets for benchmarking, comparative analysis, and validation of computational pipelines. In this context, the SARS-CoV-2 Ligands Database presented in this work may serve not only as a resource for exploring experimentally resolved interaction patterns, but also as a reference dataset for testing, calibrating, and validating structure-based prediction, docking, and interaction modeling approaches in future studies. Overall, the SARS-CoV-2 Ligands Database offers a robust, well-organized, and comprehensive resource that synthesizes the currently available structural knowledge on spike–ligand interactions. Its design ensures compatibility with future updates, including newly resolved structures, variant-specific spike domains, and expanded ligand classes. As SARS-CoV-2 continues to evolve, tools that integrate detailed structural information with functional context will be essential for anticipating antigenic drift, mapping conserved therapeutic epitopes, and guiding rational design of broad-spectrum countermeasures [34,35]. The present database provides an important foundation for such efforts and is expected to serve as a valuable resource for structural biologists, immunologists, computational modelers, and developers of antivirals and vaccines.

The in-depth analysis of RBD–antibody complexes further demonstrates the utility of the database for functional annotation. The structural analysis of RBD-antibody complexes revealed that the majority of experimental structures correspond to antibodies that recognize epitopes within or near RBM. Consistent with established classifications, most antibodies in our dataset belonged to class 1, with substantially fewer examples of class 2, class 3, and class 4 antibodies (41, 4, 2, and 9 structures, respectively). These findings align with the known epitopes that directly overlap the ACE2-binding site [36,37], which drive the strong representation of class 1 antibodies in structural studies. As shown in Figure 4, classes 1 and 2 both target epitopes within the RBM; however, class 1 antibodies typically align directly with ACE2-contact residues, whereas class 2 antibodies bind adjacent RBM regions. In contrast, antibodies in classes 3 and 4 bind distal surfaces of the RBD that do not directly overlap the ACE2-binding ridge and likely act through steric hindrance or destabilization rather than by directly blocking receptor engagement.

Detailed mapping of the antibody footprints showed distinct patterns of residue usage across antibody classes (Figure 5). Motif extraction revealed clusters of residues with high (>50%) occurrence frequency within each class. For classes 1 and 2, these motifs markedly overlapped with canonical RBM residues, including L455, F456, Y473, A475, and Y505, reflecting the central role of the RBM in neutralizing antibody recognition [38]. These findings agree with prior structural analyses demonstrating that neutralizing activity is predominantly mediated by antibodies that directly occlude the ACE2-binding interface [39]. In addition to these expected RBM residues, several additional positions outside the classical RBM definition were frequently captured across the dataset, suggesting that some antibodies engage structurally adjacent loops or secondary structural elements to enhance binding affinity.

The binding motifs of classes 3 and 4 highlighted a distinct architectural principle. Although these antibodies do not directly overlap with the ACE2-binding loops, several residues from their motifs occupy regions close to the RBM and occasionally extend toward ACE2-contacting areas [19]. This supports the hypothesis that classes 3 and 4 may contribute to neutralization through partial steric interference with ACE2 binding or by restricting conformational transitions required for efficient receptor engagement. The concentration of binding residues near the 420–440 and 500–520 segments for these classes suggests that these regions of the RBD serve as auxiliary neutralization platforms despite not constituting the primary ACE2-binding surface [40].

A further limitation of the present analysis concerns antibody classes represented by a very small number of available structures. In such cases, the application of frequency-based thresholds for motif definition lacks statistical robustness, as nominal cutoffs (e.g., presence in ≥50% of complexes) do not convey meaningful significance when the sample size is extremely limited. For example, in antibody class 3, which is represented by only two structures, formal statistical resampling approaches such as bootstrapping were considered but deemed unreliable and potentially misleading due to insufficient data. Consequently, motif identification for sparsely populated classes is presented in a descriptive and exploratory framework only. These motifs should be interpreted as indicative of preliminary structural recurrence rather than as statistically validated interaction signatures.

A notable outcome of the motif analysis is the identification of tyrosine-containing motifs, such as YNYL, YFPL, YQAG, YKLP, and YSVL, consistent with the consensus YXXØ pattern across all four antibody classes. The YXXØ pattern (a Y followed by two variable amino acids (XX) and then a hydrophobic amino acid (Ø)), is classically associated with regulatory functions in membrane-associated proteins, including internalization and signal transduction [41,42]. Although the RBD is not itself membrane-embedded, the recurrent presence of these tyrosine-centered motifs within antibody-interacting regions may indicate their structural prominence and accessibility on the RBD surface. It is plausible that the enrichment of tyrosine-based motifs contributes to antibody recognition by providing aromatic residues that facilitate stable hydrophobic or π–π interactions [43]. Within the context of ACE2 competition, the overlap of these motifs with key RBM positions suggests that their incorporation into antibodies may be sufficient to disrupt receptor binding.

In addition to mapping RBD residues involved in antibody binding, we examined the amino acid composition of antibody binding regions from the antibody side (Figure 6). Analysis of single-chain antibodies demonstrated that interactions with the RBD are dominated by tyrosine (Y), serine (S), and glycine (G) residues. This finding is consistent with the well-established role of tyrosine in antigen recognition, as it frequently participates in hydrogen bonding, π stacking, and hydrophobic interactions that stabilize antibody–antigen complexes [44]. Serine and glycine, in turn, provide conformational flexibility within complementarity-determining regions (CDRs), enabling antibodies to access structurally diverse epitopes on the RBD surface [45]. Together, this profile underscores the chemical and structural principles that shape antibody paratopes targeting the SARS-CoV-2 RBD.

The combined residue-level and motif-level analyses highlight both the shared and class-specific principles governing antibody engagement of the RBD. For class 1 and 2 antibodies, recognition is primarily driven by conserved RBM hotspots that directly overlap the ACE2 interface, while class 3 and 4 antibodies rely on auxiliary surfaces and partially overlapping regions to exert steric or conformational constraints on receptor binding. In turn, the identified tyrosine-based motifs and the amino acid distribution within the antibody–RBD binding sites may further assist in the development of new antibody engineering strategies.

## 5. Conclusions

Here, we carried out in silico ligand-based screening of PDB, collected data and presented their in the classified SARS-CoV-2 Ligands Database (http://oka.protres.ru/SARS-CoV-2Ligands, accessed on 4 January 2026), categorized based on the number of ligands and structural components of the Spike glycoprotein. Antibodies (one chain/ single name of ligand) of RBD were divided into four classes differing in position relative to the RBD. Antibodies in RBD class 1 and class 2 are more likely to lose their neutralizing activities as the viral epitopes they target are more prone to mutate. The dataset and analytical framework developed here provide a valuable resource for future structure-guided antiviral discovery. By enabling rapid assessment of ligand–spike interactions and informing rational antibody design, this work contributes to the broader effort to design countermeasures robust to ongoing SARS-CoV-2 diversification.

## Figures and Tables

**Table 1:** Distribution of SARS–ligand structures. The structures are divided into groups according to their position along the polypeptide chain (Figure 1) with corresponding names: NTD—N-terminal domain, RBD—receptor-binding domain, CTD2—C-terminal domain 2, FP—fusion peptide, CH—central helix region, HR1—heptad repeat 1, HR2—heptad repeat 2, CD—connector domain, TM—transmembrane domain. Full-length SARS corresponds to the position 1–1273 a.a. residues. All molecules of the complex (chains) were considered as ligands. The number of all available structures equals to 1797.

Domain Name	Number of Structures	Without Ligands	One Ligand	Two Ligands	Three Ligands	More Than Three Ligands
full-length SARS	977	341	231	368	5	32
full-length +TM	79	27	16	25	3	8
NTD+RBD+CTD1+CTD2	5	0	2	0	0	3
NTD	24	2	1	15	1	5
RBD	628	6	175	317	30	100
CTD2	1	1	0	0	0	0
FP	11	1	1	5	3	1
CH	4	0	0	1	0	3
HR1	24	4	11	9	0	0
HR2+TM	37	4	17	14	2	0
CD	6	0	0	1	5	0
FP-HR1	1	1	0	0	0	0
total	1797	387	454	755	49	152

## Data Availability

The original contributions presented in this study are included in the article/Supplementary Material. Further inquiries can be directed to the corresponding author.

## Supplementary Materials

The following supporting information can be downloaded at: https://www.mdpi.com/article/10.3390/biom16010163/s1, Table S1: List of single-chain antibodies of the RBD domain of SARS-CoV-2 (http://oka.protres.ru/SARS-CoV-2Ligands, accessed on 8 December 2025) and their division into classes 1–4 in accordance with their order relative to the RBD.

## Abbreviations

The following abbreviations are used in this manuscript:

SARS-CoV-2	Severe acute respiratory syndrome coronavirus 2
ACE2	Angiotensin-converting enzyme 2
RBD	Receptor-binding domain
TMPRSS2	Transmembrane serine protease 2
NTD	N-terminal domain
CTD1	C-terminal domains
FP	Fusion peptide
HR1	Heptad repeat 1
HR2	Heptad repeat 2
RBM	Receptor-binding motif
CTDs	C-terminal domains
FPPR	Fusion peptide proximal region
CD	Connector domain
6HB	Six-helix bundle
nAbs	Neutralizing antibodies
SH	Stem helix
CoV-RDB	Coronavirus Antiviral & Resistance Database
PDB	Protein Data Bank
SQL	Structured Query Language
EM	Electron microscopy
CDRs	Complementarity-determining regions
